# Response to: Comment on Genetic Ancestry-Specific Molecular and Survival Differences in Admixed Breast Cancer Patients

**DOI:** 10.1097/AS9.0000000000000424

**Published:** 2024-04-26

**Authors:** Alexandra E. Hernandez, Brandon Mahal, Aristeidis G. Telonis, Maria Figueroa, Neha Goel

**Affiliations:** * From the Division of Surgical Oncology, Department of Surgery, University of Miami Miller School of Medicine, Miami, FL; † Sylvester Comprehensive Cancer Center, University of Miami Miller School of Medicine, Miami, FL; ‡ Department of Radiation Oncology, University of Miami, Miami, FL; § Department of Biochemistry and Molecular Biology, University of Miami Miller School of Medicine, Miami, FL; ‖ Department of Human Genetics, University of Miami Miller School of Medicine, Miami, FL; ¶ Department of Medicine, University of Miami Miller School of Medicine, Miami, FL.

The points raised in the letter to the editor highlight important considerations and ongoing discussions in the field of genetic ancestry research, along with known limitations of large genomic databases such as The Cancer Genome Atlas (TCGA). The objective of *Genetic Ancestry-Specific Molecular and Survival Differences in Admixed Breast Cancer Patients* was to build on foundational studies in the literature that have evaluated the association between genetic ancestry and breast cancer subtype, recurrence, and survival using TCGA.^1^ However, a critical gap in these studies is that patients were characterized as “genomic Black” (defined as ≥50% African ancestry) or “genomic White” (defined as ≥90% European ancestry).^2^ A study by Huo and colleagues^2^ discovered molecular features associated with breast cancer subtypes and recurrence varied by Black and White genetic ancestry categories, concluding that these differences may be partly caused by germline genetic variants. However, since categorization may limit the discovery of potential molecular differences in key admixed populations, the study by Telonis and colleagues^1^ fills a critical gap in the literature by evaluating genetic ancestry and its association with breast cancer intrinsic subtypes and molecular differences using *incremental changes* in ancestry in an *admixed* population of women from TCGA, thus moving toward a more inclusive approach to study genetic ancestry in admixed populations where neither West African nor European ancestry is greater than 90%.

Our objective in including admixed populations is to veer the discussion away from racial essentialism. The importance of our work is the emphasis on disentangling genetic ancestry from self-identified race and ethnicity and understanding the limitations of current national genetic ancestry databases, such as TCGA, which do not include social determinants of health. Telonis et al^1^ specifically state that it is “imperative to conduct health disparities research that accounts for the social, ecological, political and/or historical exposures within a neighborhood that can impact health outcomes.” Admixture analysis gives us additional opportunities to detect genes that influence disease states by detecting major allele frequency differences in ancestrally divergent populations within an admixed population.^3,4^ The increasing admixture in the United States and elsewhere makes the use of genetic ancestry along with self-identified race and ethnicity even more vital. A recent study by Martini and colleagues highlights how the inclusion of admixed patient groups in genomic research advances the field instead of using self-identified race and ethnicity as a proxy for genetic ancestry or global ancestry cutoffs to define a patient group, which misses a large amount of multiethnic and multiracial patients that are a result of colonialism, structural racism, and population history, along with genomic backgrounds of admixed groups. This is particularly relevant as Telonis and colleagues show vast diversity in genetic ancestry percentages, even among patients within the same self-identified race (Asian, Black, and White) and ethnicity (Hispanic) groups.^2^

The importance of studying both genetic inferred ancestry and self-identified race and ethnicity cannot be overstated. Genetic inferred ancestry reflects population history, providing background information about genetic variation that is important in understanding genomic associations with diseases. However, self-identified race and ethnicity are sociopolitical constructs and not ones based on biology. Racial and ethnic groups do not provide a genetic basis for differences in cancer outcomes, and genetic ancestry cannot be used as a proxy for race or ethnicity, as it does not account for the important sociopolitical and cultural aspects of these measures. Both self-identified race and ethnicity and genetic ancestry can reflect biological and genomic differences in distinct ways with clinical significance. While self-identified racial and ethnic groups do not provide a genetic basis for differences in cancer outcomes, many studies have identified associations between self-identified race and ethnicity and breast cancer subtypes, particularly triple-negative breast cancer^5–9^ (TNBC), along with race-group distinctions in genomic differences such as single-nucleotide variants,^10–12^ somatic mutations,^13,14^ copy-number variations,^15^ and DNA methylation differences.^16^ To build on these findings, a study by Davis and colleagues^5^ identified African ancestry-specific gene expression differences in TNBC tumors in admixed African American women compared with European ancestry women. While they did find some correlations with self-identified race-associated gene networks, almost half (48.1%) of the African ancestry-associated genes were distinct from the self-identified race-associated genes.^5^ This highlights the distinct genetic influences of genetic ancestry beyond self-identified race and suggests that genetic factors tied to West African ancestry may contribute to a more aggressive breast cancer subtype. Additionally, their work found significant associations between African ancestry and the TNBC immune profile, potentially explaining the varied clinical responses among different racial groups.

Combined, these studies suggest that there may be genetic underpinnings beyond the sociopolitical reasons for disparities. While some of these may be germline or somatic mutations, we also must recognize the (epi)genomic consequences of social adversity faced by mostly minority populations, such as self-identified Black and Hispanic patients. As a result of structural racism and discrimination, communities of color experience considerably higher levels of social adversity through discriminatory practices and mutually reinforcing systems of racial inequality (structural racism), including housing, education, employment, health care, criminal justice, income/poverty, and the built environment. Therefore, some of these genomic associations suggested in disparities studies may be more related to social adversity and stress-related epigenomic changes secondary to socioeconomic disadvantage.^17–19^ This highlights that genetic ancestry cannot be used as a proxy for race or ethnicity, as it does not account for these measures’ important sociopolitical and cultural aspects.

With respect to Olsen and colleagues’ comments regarding causality, Telonis and colleagues do not state causality in their findings between ancestry and intrinsic breast cancer subtypes and outcomes.^20^ The field of genetic ancestry research is still attempting to identify frameworks to study ancestry-related biology that also accounts for social, behavioral, and environmental factors along the causal pathway,^21^ at least 2 directed acyclic graphs (DAGs) representative of 2 causal theories explaining (1) biomedical and (2) social causes of mortality.^21^ Moreover, the authors acknowledged that other criteria for establishing causation, such as temporality, are possible through DAGs that include time-varying relationships, although this type of data is rarely available. As stated by Iyer and colleagues, the objective of their DAGs is to capture the strongest causal assumptions backed by literature and clinical knowledge and to create these DAGS with accessibility to these data elements in mind.^21^

Olsen and colleagues expressed interest in findings showing that the “observed gene expression works in opposite directions in luminal and basal cancers.” And continue by stating that “If both continental ancestry groups can both increase and decrease expression of the same genes, it seems to imply that something else mediates the association between gene expression and breast cancer.” We want to point out well-established dependencies on the biological context of breast cancer. Different prediction analysis of microarray 50 subtypes are considerably distinct, with different molecular characteristics, different responses to treatments, and, overall, different cellular and molecular biology.^22,23^ Thus, findings that ancestry correlates with the same genes differently in different disease contexts (prediction analysis of microarray 50 subtypes) are not surprising. Tumor suppressor genes, including coding and noncoding ones, in one context, maybe more oncogenic than in another context.^24–27^ To elucidate the mechanisms by which genetic ancestry is associated with gene expression would require extensive experimentation without readily available models of genetic ancestry, thus, exceeding the scope of our original study.

Overall, Telonis and colleagues fill a critical gap in the genetic ancestry and breast cancer literature by studying ancestry as a continuous variable in an admixed population. As the authors clearly state in their discussion, when conducting such research, it is important to understand the limitations of current national genetic ancestry databases such as TCGA, which do not include social determinants of health or information on structural racism.^28^ Our approach to disentangling the concepts of genetic ancestry and race and ethnicity includes understanding the impact of social determinants of health, neighborhood contextual-level factors, and structural racism to inform a “translational epidemiologic” approach, pioneered in part by Goel and colleagues to study disparities.^29–31^ To accomplish this, our team at the University of Miami established the Miami Breast Cancer Disparities Study, a prospective longitudinal cohort study that integrates both genomic and nongenomic factors to comprehensively study breast cancer disparities after adequately controlling for confounders not readily available in large genomic databases such as TCGA. Utilizing this genomic-epidemiologic cohort, Goel and colleagues discovered that independent of West African ancestry, women living in a low-income neighborhood had higher odds of TNBC, suggesting that factors associated with one’s neighborhood may also impact breast cancer subtype development.^30^ The authors, therefore, proposed that the field of genetic ancestry research take a “translational epidemiologic” framework to understand gene-environment interactions and social (epi)genomics to understand how both sociopolitical and cultural concepts of race and ethnicity and genetic ancestry, which may also reflect social factors, influence breast cancer outcomes.^29^

As stated by Telonis and colleagues, “future large-scale studies must take a translational epidemiologic approach to integrate multi-omic sequencing, genetic ancestry, neighborhood socioeconomic status, and additional molecular features associated with breast cancer subtype to improve outcomes in historically marginalized individuals.”^1^ Utilizing this approach can advance precision oncology as it will allow for improved methods of targeted, neighborhood-based patient risk stratification, screening, and other cancer control interventions.
